# Cerebral lactate dynamics across sleep/wake cycles

**DOI:** 10.3389/fncom.2014.00174

**Published:** 2015-01-14

**Authors:** Michael J. Rempe, Jonathan P. Wisor

**Affiliations:** ^1^Mathematics and Computer Science, Whitworth UniversitySpokane, WA, USA; ^2^Department of Integrative Physiology and Neuroscience, College of Medical Sciences, Washington State University SpokaneSpokane, WA, USA

**Keywords:** sleep, lactate, mathematical modeling, metabolism, optimization, process S, slow wave

## Abstract

Cerebral metabolism varies dramatically as a function of sleep state. Brain concentration of lactate, the end product of glucose utilization via glycolysis, varies as a function of sleep state, and like slow wave activity (SWA) in the electroencephalogram (EEG), increases as a function of time spent awake or in rapid eye movement sleep and declines as a function of time spent in slow wave sleep (SWS). We sought to determine whether lactate concentration exhibits homeostatic dynamics akin to those of SWA in SWS. Lactate concentration in the cerebral cortex was measured by indwelling enzymatic biosensors. A set of equations based conceptually on Process S (previously used to quantify the homeostatic dynamics of SWA) was used to predict the sleep/wake state-dependent dynamics of lactate concentration in the cerebral cortex. Additionally, we applied an iterative parameter space-restricting algorithm (the Nelder-Mead method) to reduce computational time to find the optimal values of the free parameters. Compared to an exhaustive search, this algorithm reduced the computation time required by orders of magnitude. We show that state-dependent lactate concentration dynamics can be described by a homeostatic model, but that the optimal time constants for describing lactate dynamics are much smaller than those of SWA. This disconnect between lactate dynamics and SWA dynamics does not support the concept that lactate concentration is a biochemical mediator of sleep homeostasis. However, lactate synthesis in the cerebral cortex may nonetheless be informative with regard to sleep function, since the impact of glycolysis on sleep slow wave regulation is only just now being investigated.

## 1. Introduction

It has been known for some time that the brain's utilization of glucose declines dramatically during SWS relative to wake. It is widely thought that this decline in cerebral metabolism is a central function of sleep. In humans, glucose uptake in the brain is reduced during slow wave sleep (SWS) relative to wake (Kennedy et al., 1982; Buchsbaum et al., 1989; Maquet et al., 1990). In rodent tissues analyzed either by microdialysis/high performance liquid chromatography (Kalinchuk et al., 2003; Wigren et al., 2009) or post mortem biochemical assay (Van and Brine, 1970; Reich et al., 1972), brain concentration of lactate, the product of glycolytic metabolism of glucose, declines during sleep relative to wake. Recently, indwelling biosensors have been utilized to measure the concentration of lactate in the cerebral cortex of rodents with a temporal precision not previously possible (Clegern et al., 2012; Dash et al., 2012; Naylor et al., 2012; Wisor et al., 2013). These studies have demonstrated that cerebral lactate concentration declines during SWS relative to wake in rodents. More specifically, we have shown that cerebral lactate concentration varies as an inverse function of SWA in the cerebral cortex (Wisor et al., 2013) indicating that lactate concentration may be a biochemical manifestation or regulator of slow wave activity (SWA).

SWA in the electroencephalogram varies as a function of sleep/wake cycles. SWA increases as a function of prior time spent awake and decreases as a function of prior time spent asleep. This very reliable predictive relationship has been documented across a broad range of mammalian species (Borbely, 1982; Trachsel et al., 1988; Franken et al., 1991; Tobler et al., 1993) and has been modeled mathematically by a set of saturating exponential equations known as process S. Sleep state-dependent changes in the concentrations of neuromodulatory molecules, sleep substances, in the cerebral cortex may underlie the sleep state-dependent dynamics of SWA. If lactate is a sleep substance its concentration would be expected to vary inversely with SWA in the cerebral cortex as a function of sleep and wake states. We (Clegern et al., 2012; Wisor et al., 2013) and others have described the short-term dynamics of lactate sensor output as a function of sleep/wake state transitions and EEG SWA. The previously reported data do not address the possibility that lactate concentration exhibits longer-term state-dependent dynamics in parallel with the homeostatic dynamics of EEG SWA. In order to gauge this possibility, it was necessary to describe lactate dynamics on the minutes-to-hours time scale on which EEG slow wave dynamics have previously been be described mathematically with a general homeostatic model known as “Process S” (Borbely, 1982; Trachsel et al., 1988; Franken et al., 1991; Tobler et al., 1993).

Here, we applied a general homeostatic mathematical model conceptually based on Process S to model lactate dynamics *in vivo* in the cerebral cortex. According to the model, lactate concentration increases toward an upper asymptote as a function of time spent awake or in rapid eye movement sleep and decreases toward a lower asymptote as a function of time spent in SWS. Additionally, we applied a well-known method of parameter optimization, the Nelder-Mead algorithm, in modeling the homeostatic dynamics of SWA and lactate concentration. This algorithm, in contrast to the previously used “brute force” methods (Borbely, 1982; Trachsel et al., 1988; Franken et al., 1991; Tobler et al., 1993) (where every possible combination of the parameters within the parameter space is investigated), systematically restricts the parameter space in which iterations of the model are run, thereby reducing computational time by orders of magnitude relative to the brute force method. The goals of the current study are (1) to quantify the sleep/wake dependent dynamics of cerebral lactate concentration in a common mouse strain using a general homeostatic model akin to Process S and (2) to demonstrate a computationally efficient mathematical method for optimizing the values of the model parameters. The results show that lactate varies as a saturating exponential function of state across sleep/wake cycles. We compare the parameter values obtained in modeling lactate sensor values to those obtained in modeling EEG SWA dynamics.

## 2. Methods

### 2.1. Experimental subjects

All procedures adhered to National Research Council guidelines (Institute of Laboratory Animal Resources NRC 1996) and were approved by the institutional animal care and use committee of Washington State University. Male mice of the C57BL/6J strain were used in these experiments. They were purchased from Jackson Laboratories (Bar Harbor, ME; strain # 664). At the time of surgical implantation, mice weighed 26.1 g on average and were 91 days old. Animals were housed in an LD12:12 cycle with unrestricted access to standard laboratory chow and water. Experimentation was completed within 3 weeks of surgical implantation.

### 2.2. Surgical preparation of subjects

Surgery was performed as described previously (Clegern et al., 2012; Wisor et al., 2013) under isoflurane anesthesia. Mice were surgically equipped for bilateral referential fronto-parietal EEGs and neck electromyograms (EMGs). Frontal EEG electrode locations were 1.5 mm lateral to the midline and 1 mm anterior to bregma. The parietal EEG electrode location was 1.5 mm left of midline and 2 mm anterior from lambda. A guide cannula was placed in the left frontal cerebral cortex 1.1 mm anterior and 1.65 mm lateral from bregma. A dummy stylet was placed in the guide cannula until the day of experimentation, 10–14 days after surgery. Animals were subjected to 48 h of spontaneous sleep recording under standard conditions (Wisor et al., 2011, 2013), interrupted by a 3-h gentle handling sleep deprivation session from ZT 3 to ZT 6 in the second light/dark cycle.

### 2.3. Data collection and analysis

Lactate was measured using a lactate oxidase based biosensor implanted in the frontal cerebral cortex as described elsewhere (Clegern et al., 2012; Wisor et al., 2013). This technique was first published in 1997 (Hu and Wilson, 1997) and has since been refined and commercialized by Pinnacle Technologies (Pinnacle technologies part #7004-Lactate). The sensing mechanism consists of a platinum-iridium electrode surrounded by a layer of lactate oxidase molecules. Metabolism of lactate by lactate oxidase produces hydrogen peroxide, which produces a current in the platinum-iridium electrode. The current at the sensing electrode is proportionate to the concentration of the substrate for the lactate oxidase enzyme (lactate, the product of glycolysis). Current is monitored at a sampling rate of 400 Hz providing a moment-by-moment estimate of lactate concentration in the cerebral cortex. Current was averaged across all values (*n* = 4000) within each 10-s epoch of data analyzed.

On the day of experimentation, the lactate sensor was pre-calibrated as described elsewhere (Clegern et al., 2012; Wisor et al., 2013). Approximately 8 h into the light portion of the LD12:12 cycle, the dummy stylet was removed from the guide cannula and the lactate sensor was inserted into the guide cannula. The uninsulated, enzymatically active portion of the sensor was embedded at a depth of 1 mm in the cerebral cortex. This depth targets the deeper layers of cerebral cortex, although placement was not verified histologically. The animal was placed into a cylindrical cage where it remained throughout the duration of the recording session. EEG, EMG and lactate biosensor current were monitored continuously at 400 Hz for 40–48 h thereafter. During some recordings the lactate sensor required several minutes to equilibrate, and this equilibration time varied from recording to recording. Therefore, before fitting a model to lactate data we discarded lactate data before the second SWS episode of at least 1 min. Lactate data were smoothed using a median filter of width 3 epochs to eliminate artifacts.

### 2.4. Mathematical model for EEG activity in the delta frequency range

To quantify the temporal dynamics of EEG activity we employed a general homeostatic model, similar to that used in the two-process model of sleep dynamics (Borbely, 1982; Daan et al., 1984). This type of model has previously been used to quantify EEG SWA dynamics in genetically diverse mouse strains (Franken et al., 2001). Delta power (1–4 Hz) was quantified within each 10-s epoch by fast Fourier transform of the EEG as described elsewhere (Wisor and Clegern, 2011). The time course of the SWA (Process S) was calculated assuming that S increases exponentially toward an upper asymptote (UA) during wake and REM, and decreases exponentially toward a lower asymptote (LA) during SWS. Mathematically, the update step for S is given as follows:

(1) $$S_{t+1}\;=UA-(UA-S_{t})e^{\frac{-\Delta t}{\tau_{i}}}\text{ Wake or REM}$$

(2) $$S_{t+1}\;=LA+(S_{t}-LA)e^{\frac{-\Delta t}{\tau_{d}}}\text{ SWS}$$

Where τ_*i*_ and τ_*d*_ are the time constants of the increasing and decreasing exponential functions, respectively, *S_t_* and *S*_*t*+1_ are the values of *S* at consecutive 10 s epochs, and Δ*t* is the time step (10 s), *UA* is a constant asymptote that *S* approaches from below during wake or REM and *LA* is an asymptote that *S* approaches from above during SWS. These two difference equations can be written more compactly as a single differential equation:

(3) $$\frac{dS}{dt}=\sigma \left(\frac{-1}{\tau_{d}}\right)(S-LA)+(1-\sigma )\left(\frac{-1}{\tau_{i}}\right)(S-UA)$$

where σ = 1 during an epoch scored as SWS and σ = 0 otherwise. To determine the values of the parameters *UA* and *LA* we constructed one histogram of delta power for 10 s epochs scored as REM, and another histogram of delta power for 10-s epochs scored as SWS. The 99% level for the SWS distribution was chosen as the value of *UA* and the intersection of the SWS and REMS histogram curves was chosen as *LA* following (Franken et al., 2001). To initialize *S*, the simulation was run once for 24 h starting at the average of *UA* and *LA*. Then the simulation was started over using the value obtained at *t* = 24 as the new starting value for *S*. We found that the choice of starting value for *S* for this 24 h initialization run did not affect the value of *S* at *t* = 24 as the curve starting at *UA* and the curve starting at *LA* quickly converged to the same curve. The only remaining free parameters in the model are τ_*i*_ and τ_*d*_. The optimal values of these two parameters were found in two ways: a brute force approach and a fast iterative method called Nelder-Mead also known as the simplex method. These two methods are described in more detail in Section 2.6.

### 2.5. Mathematical model for lactate dynamics

Since cerebral lactate concentration rises during wakefulness and REM and falls during episodes of SWA (Wisor et al., 2013), we employed the same type of model for lactate concentration:

(4) $$L_{t+1}\;=UA_{t}-(UA_{t}-L_{t})e^{\frac{-\Delta t}{\tau_{i}}}\text{ Wake or REM}$$

(5) $$L_{t+1}\;=LA_{t}+(L_{t}-LA_{t})e^{\frac{-\Delta t}{\tau_{d}}}\text{ SWS}$$

The model for the wake-sleep temporal dynamics of lactate (which we term Process L) is identical to that of Process S except that the upper and lower asymptotes, (*UA* and *LA*) respectively, are functions of time rather than constants. This is done to account for the changes in the lactate signal due to the gradual decay in the output of the lactate sensor over the course of the recording. To compute *UA*(*t*) and *LA*(*t*) we constructed a histogram of the lactate signal in a 2-h moving window throughout the entire recording. The 90% level of these histograms was chosen as *UA* and the 10% level was chosen as *LA*. An example of *UA* and *LA* are shown in **Figure 2B**.

### 2.6. Optimizing the choices of τ_*I*_ and τ_*D*_

**Figure 1:**
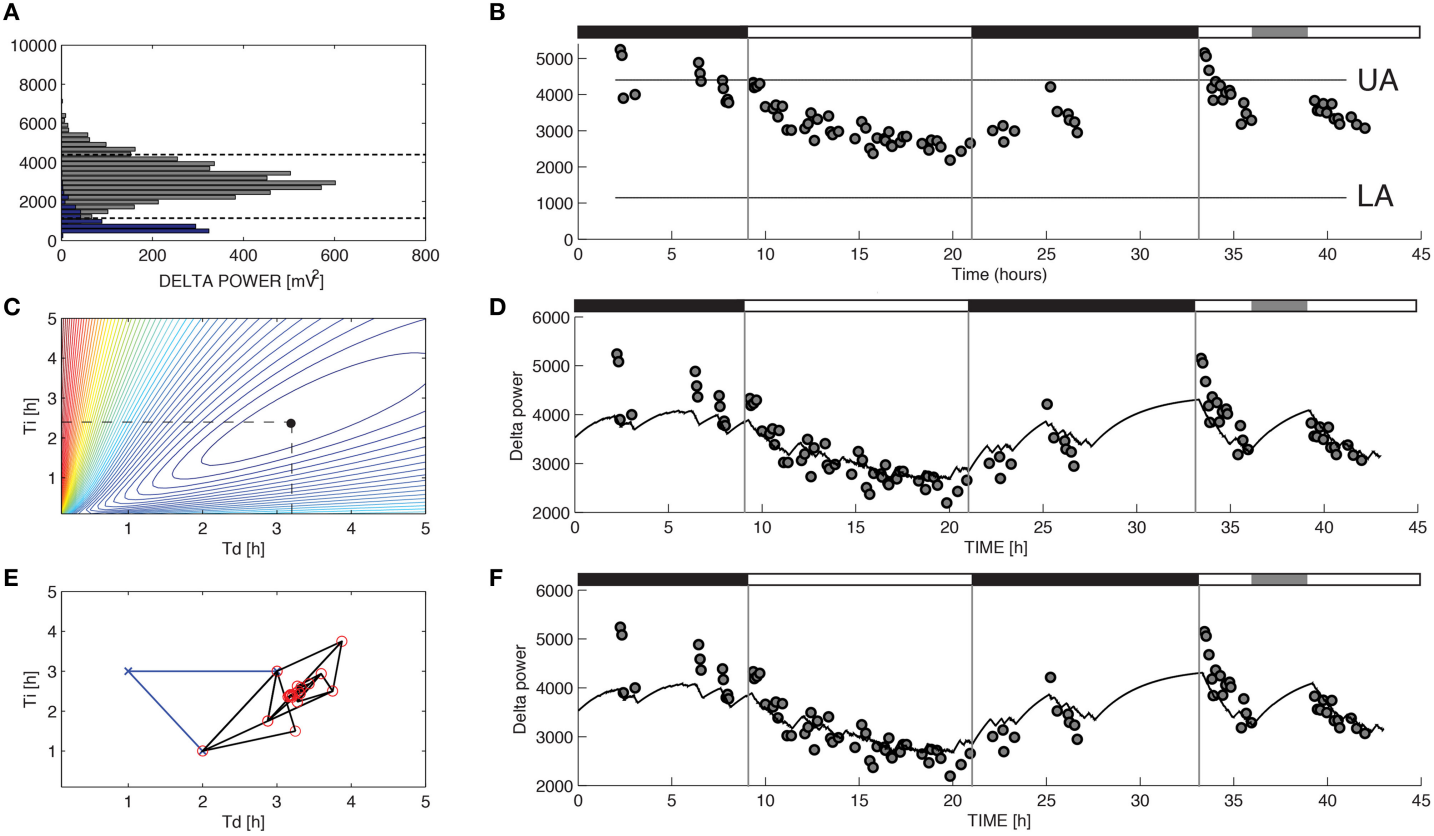
**Parameter estimation for Process S in a representative recording from a C57BL/6 mouse**. The variable S was assumed to increase toward an upper asymptote UA during 10-s epochs of wakefulness and rapid eye movement sleep and decrease toward a lower asymptote LA during 10-s epochs of slow-wave sleep (SWS) according to Equations 1 and 2. **(A)** UA and LA were constructed using the relative frequency histogram of delta power for 10-s epochs scored as R or SWS during the recording. The 99% level of the SWS histogram was chosen as the upper asymptote (UA) and the intersection of the histogram curves for SWS and R was chosen as the lower asymptote (LA). **(B)** The data used to choose the optimal values of τ_*i*_ and τ_*d*_ were the median values of delta power reached during 5-min segments in which at least 90% of the epochs were scored as SWS. **(C)** To determine the optimal values for the parameters τ_*i*_ and τ_*d*_ in brute force fashion we performed an exhaustive search over a reasonable range of values for these two parameters and computed the sum of squares error for each combination. **(D)** Best fit of the model to the data from **(B)** using the optimal parameters found from exhaustive search (τ_*i*_ = 2.39 and τ_*d*_ = 3.18). **(E)** Successive guesses for the optimal choices of τ_*i*_ and τ_*d*_ using the Nelder-Mead method. The iterative method converges to (τ_*i*_ = 2.39 and τ_*d*_ = 3.18) in just 45 calculations. **(F)** Best fit of the model to the data from **(B)** using the optimal parameters found from Nelder-Mead. Black horizontal bars on the tops of **(B,D,F)** indicate the 12 h dark periods and the gray horizontal bar indicates when sleep deprivation occurred.

To choose the optimal values of τ_*i*_ and τ_*d*_ for both process *S* and process *L* we took two approaches. First we used a “brute force” approach: we chose a reasonable range of potential values for each time constant and we iterated over every possible combination of the two parameters. For process *S* these ranges were the same as those used in Franken et al. (2001), for process *L* we established a range of potential combinations based on preliminary analyses which indicated that process *L* has faster dynamics than process *S*. For each combination of parameter values the simulation was run and the error between the model and the data was computed. For the case of process *S* the model was compared to median delta power in 5-min segments containing at least 90% SWS (Figure 1B) following (Franken et al., 2001). In the case of lactate, the model was compared to the lactate value at every epoch. In both process *S* and process *L*, the mean sum of squares error was computed for every possible combination of the τ values and the combination yielding the lowest error was chosen as the optimal choice. While this method is guaranteed to find the optimal choices for the time constants (as long as the optimal combination lies within the ranges chosen for each parameter), it results in a huge number of calculations and the computing time can be prohibitive, even when optimizing only two unknown parameters. Previous applications of Process S required nearly 40,000 runs of the model for each data set to optimize τ_*i*_ and τ_*d*_ (Franken et al., 2001). Computing time for this method, using the same number of iterations as in Franken et al. (2001), is shown in Table 1.

**Table 1:** **Optimal parameter values and running times for the Nelder-Mead (NM) and brute force (BF) methods**.

	**τ_*i*_(h)**	**τ_*d*_(h)**	**Running time (s)**	**Residuals (raw)**	**Residuals (normalized)**
SWA NM	2.00 (0.06)	2.91 (0.06)	2.75 (0.02)	981.56	0.1008
SWA BF	1.99 (0.06)	2.90 (0.06)	362.79 (0.24)	981.63	0.1008
Lactate NM	0.94 (0.06)	0.34 (0.02)	2.78 (0.03)	0.6567	0.0764
Lactate BF	0.91 (0.06)	0.33 (0.02)	735.16 (3.11)	0.6575	0.0764

*Values are means with standard errors in parentheses*.

In addition to using an exhaustive search to find the optimal values for the time constants we also used the Nelder-Mead method, also known as the Simplex method (Nelder and Mead, 1965; Lagarias et al., 1998). Instead of computing the error at every possible combination of the two parameters, the Nelder-Mead method begins by evaluating the error at three different points in the τ_*i*_−τ_*d*_ plane. The triangle then undergoes a series of transformations (reflections, expansions, contractions, or shrinkages) based on the values of the function at those three points. These transformations move its vertices closer to the global minimum. Each of these transformations is described in detail in Lagarias et al. (1998). The result is that instead of computing the error for tens of thousands of different combinations of the two parameters, the Nelder-Mead algorithm uses only a few dozen to rapidly find the optimal combination. Comparisons in optimal values found using brute force and Nelder-Mead, along with computing times, are shown in Table 1. All simulations were performed on a Dell Precision T7500 running Windows 7 with 24 GB of memory and an Intel Xeon 2.2 GHz. dual-core processor.

## 3. Results

For the model of SWA (Process S), optimizing the time constants with either Nelder-Mead or brute force yielded uniform τ_*i*_ and τ_*d*_ values. Mean τ_*i*_ and τ_*d*_ values differed across the two methods by less than 1 percent. Moreover, the model output closely matches the data when either brute force (Figure 1D) or Nelder-Mead (Figure 1F) is used to choose optimal values of the time constants. Process S is an exponential model that rises toward a constant upper asymptote (UA) during epochs labeled as wake or REM and falls toward a constant lower asymptote (LA) during epochs labeled as SWS (Figure 1B). This homeostatic behavior of SWS is consistent with data shown elsewhere (Franken et al., 2001) and indicates that the Process S model is appropriate for the SWA data in these experiments. To find the optimal values for τ_*i*_ and τ_*d*_, we first used a brute force approach: running the simulation and computing the error for 39,597 different combinations of τ_*i*_ and τ_*d*_ (τ_*i*_ = 1–25 h, step-size 0.12 h, and τ_*d*_ = 0.1–5 h, step-size 0.025 h). Errors for the brute-force approach, along with the optimal combination, are shown in Figure 1C. The Nelder-Mead approach to choosing parameter values required only 45 calculations to find the same optimal combination (Figure 1E).

**Figure 2:**
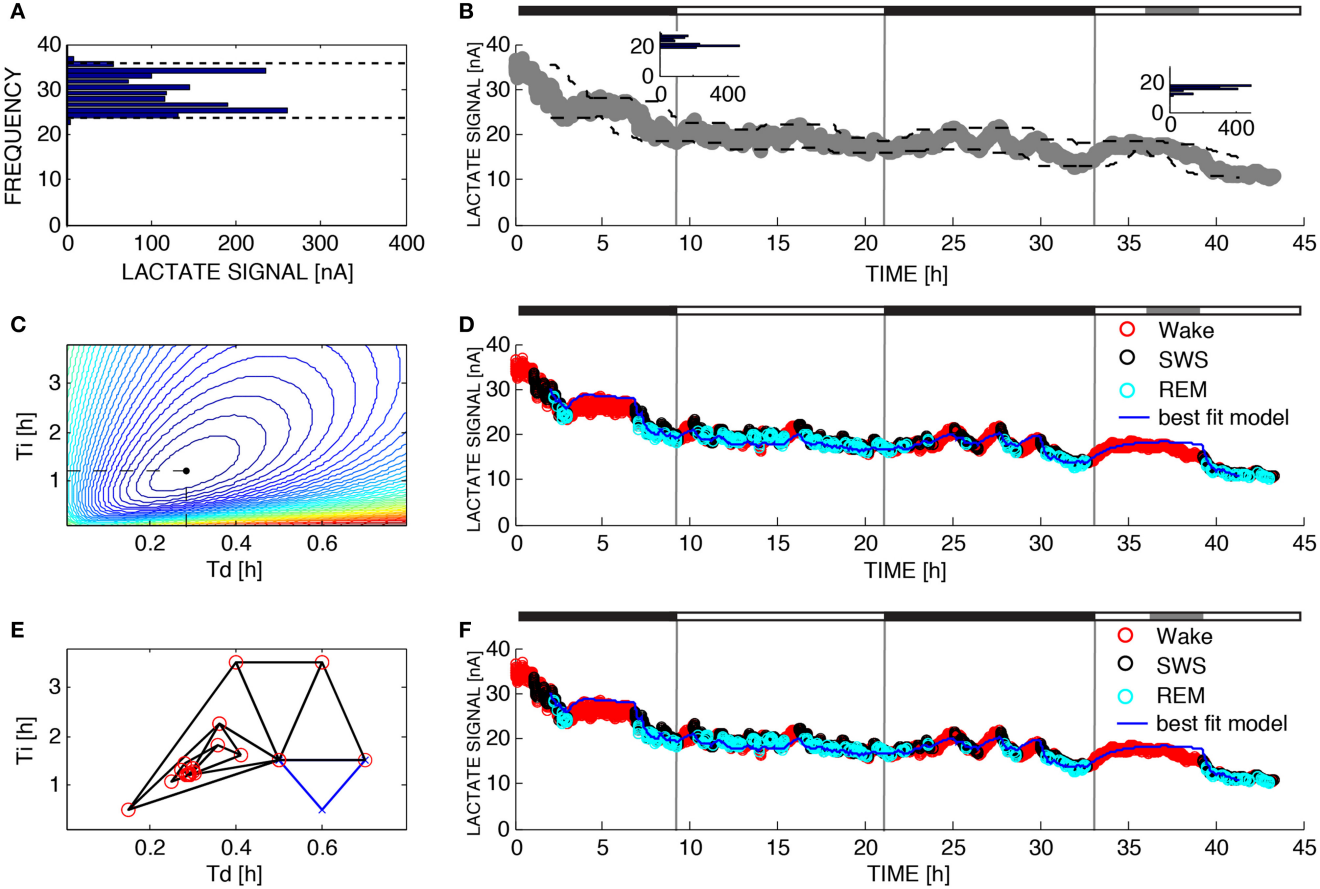
**Parameter estimation for Process L**. The variable L was assumed to increase during 10-s epochs of wakefulness and rapid eye movement sleep and decrease during 10-s epochs of SWS according to Equations 4 and 5. **(A)** UA and LA are functions of time due to the fact that the dynamic range of the lactate sensor attenuated over the 43 h of recording time. To compute UA(t) and LA(t) we constructed the relative frequency histogram of lactate for each moving 2-h window in the dataset. The 99% level of the lactate histogram was chosen as the upper asymptote (UA(t)) and the 1% level of the lactate histogram was chosen as the lower asymptote (LA(t)). **(B)** Upper left inset shows the histogram for the lactate signal at *t* = 8 h and the inset to the right shows the histogram for the lactate signal at time *t* = 38 h indicating that the 1% level and 99% level have changed over the course of the experiment. This change is taken into account in the change of the dashed lines. **(C)** To determine the optimal values for the parameters τ_*i*_ and τ_*d*_ in brute force fashion we performed an exhaustive search over a reasonable range of values for these two parameters and computed the sum of squares error for each combination. **(D)** Best fit of the model to the data from **(B)** using the optimal parameters found from exhaustive search (τ_*i*_ = 1.20 and τ_*d*_ = 0.28). **(E)** Successive guesses for the optimal choices of τ_*i*_ and τ_*d*_ using the Nelder-Mead method. The iterative method converges to (τ_*i*_ = 1.20 and τ_*d*_ = 0.28) using just 32 function evaluations. **(F)** Best fit of the model to the data from **(B)** using the optimal parameters found from Nelder-Mead. Black horizontal bars on the tops of **(B,D,F)** indicate the 12 h dark periods and the gray horizontal bar indicates when sleep deprivation occurred.

Figure 2B shows lactate data from the same recording as Figure 1 along with the upper and lower asymptotes as defined in Section 2.5 and Figure 2A shows a histogram of lactate for one 2-h window. Lactate concentration, like SWA, rises during wake or REM and declines during SWS. Figures 2D,F show the best fit of the model to the data (using brute force and Nelder-Mead respectively to choose the time constants) where each epoch of data has been color-coded for sleep state. Lactate concentration goes up during transistions from SWS-to-wake or SWS-to-REM and goes down during transitions from wake-to-SWS or REMS-to-SWS as described previously (Wisor et al., 2013). The model successfully tracks changes in lactate concentration not only during transitions between states, but also while the state is not changing. To optimize the choices of τ_*i*_ and τ_*d*_, 39,597 different simulations were run and the error was computed for each combination (Figure 2C). The optimal choices of τ_*i*_ and τ_*d*_ were the same as the optimal choices found using Nelder-Mead search, even though Nelder-Mead required only 32 runs of the model (Figure 2E).

As shown in Table 1, both the brute force method and the Nelder-Mead method return nearly the same values for the optimized parameters. The time needed to find those optimal values using the brute force approach was a factor of 132 longer than using Nelder-Mead for Process S and a factor of 264 longer for Process L. The brute force approach is faster for SWA than for lactate probably because there are fewer SWA data points to use when computing the error between the model fit and the data. The SWA simulations only use data during SWS episodes of 5 min or longer, while the lacate simulations use data from every epoch. Slight descrepancies in the optimal values obtained using Nelder-Mead vs. brute force can be explained by the fact that the brute force approach uses a fixed increment for the τ_*i*_ and τ_*d*_ values meaning the true optimum may occur in between values. The Nelder-Mead algorithm, on the other hand, makes no such restriction and therefore the values obtained with Nelder-Mead should be regarded as more accurate. Nevertheless, agreement between the two methods is quite good.

## 4. Discussion

The concentration of lactate, the product of glycolytic metabolism of glucose, is known to exhibit sleep-state dependent dynamics (Shram et al., 2002; Wigren et al., 2009; Wisor et al., 2013). The application of the indwelling enzymatic sensor to sleep studies (Clegern et al., 2012; Dash et al., 2012; Naylor et al., 2012; Wisor et al., 2013) allowed experimenters for the first time to analyze data in 10-s epochs, a level of temporal precision equivalent to that over which SWA dynamics are measured. Doing so made it possible to demonstrate a role for SWA, specifically, in determining the short-term dynamics of lactate during SWS (Wisor et al., 2013). The use of the biosensor has additionally made possible, as shown here, the application of a general homeostatic model based on Process S to describe sleep-state dependent lactate dynamics. While the previous work demonstrated a relationship between SWA and changes in lactate concentration (Wisor et al., 2013), the hypothesis that lactate concentration drives changes in SWA across sleep bouts in a monotonic fashion is not supported by the current data. Like SWA, lactate concentration declines toward a lower asymptote during SWS and rises toward an upper asymptote during wake/REMS although lactate concentration does not follow SWA activity directly and has different time constants than SWA does.

The changes in lactate in association with sleep state bear some resemblance to changes in cerebral cortical temperature across sleep/wake cycles. Both variables decline at sleep onset relative to prior wake, and increases at wake onset relative to prior SWS. This parallel raises the possibility that changes in sensor readout are influenced by cortical temperature in addition to substrate availability in the extracellular environment. To address the influence of temperature on sensor readout independently of lactate metabolism, a previous study measured the readout of an indwelling null sensor lacking lactate selectivity in parallel with the lactate sensor (Naylor et al., 2012). While the null sensor exhibited state-specific changes in current (presumably driven by temperature changes), these changes were roughly 100-fold smaller than state-specific changes in lactate sensor readout. Thus, any thermal effect is exceedingly modest relative to substrate-specific effects on sensor current. Additionally, we have demonstrated a strong predictive relationship between EEG SWA and lactate sensor readout: the rate of decline of lactate sensor readout across sleep intervals is proportional to SWA in the concurrently measured EEG (Wisor et al., 2013). Concurrent measurement of SWA and temperature in the rat cerebral cortex demonstrated no predictive relationship between SWA and brain temperature dynamics (Franken et al., 1991, 1992). This and the fact that rodent brain temperature decreases at the transition from REMS to wake (Tobler et al., 1993), while lactate sensor readout increases at the transition from REMS to wake, demonstrate independence of lactate sensor readout from brain temperature. Additional work to measure the state-dependent changes of concurrently-measured brain temperature and lactate could further strengthen this line of reasoning.

A novel approach was necessary in modeling lactate sensor data due to input signal dynamics. The dynamic range of EEG SWA remained stable over a 2-day recording, and accordingly asymptote values could be calculated and applied statically across the entirety of each recording. By contrast, the dynamic range of the lactate sensor current attenuated over the recording time. To account for this change in lactate sensor output, it was necessary to re-calculate upper and lower asymptote values in moving windows throughout the recording. Early in the analysis of these data, we attempted to detrend the data by regression analysis (data not shown). In some cases, a decaying exponential function was found to have the smallest residuals. In others, a linear exponential function was found to have the smallest residuals. We could not find a uniform regression-based de-trending model that would apply across all files. The approach that we take in the manuscript is superior in that it requires no a priori assumptions about the mathematical behavior of the long-term dynamics of the sensor readout but rather accommodates to those long-term dynamics however they behave. The reduction in the dynamic range may be biological if, for instance, cell death or gliosis occurs at the site of the sensor. Additionally, changes in sensor chemistry such as denaturation of lactate oxidase molecules or oxidation of electrodes may underlie changes in sensor readout. The changing dynamic range is a limitation inherent to the biosensor method, but the biosensor provides temporal resolution not available with the other methods that have been used to measure state-dependent changes in cerebral lactate. Sleep/wake state-dependent dynamics of lactate nonetheless remained intact across 42-h recordings despite this technical limitation: lactate sensor output continued to follow asymptotic curves in a state-dependent fashion. Additional tools, such as fluorescent analogs of glucose (Barros, 2013), and its metabolites, may provide alternative confirmation of findings reported with indwelling biosensors.

According to the two-process model, the sleep EEG is a product of the interaction of Process S with Process C, the circadian signal originating in the suprachiasmatic nucleus of the hypothalamus. Although changes in SWA are largely impacted by Process S, SWA in humans under a forced desynchrony protocol exhibits circadian modulation (Dijk and Czeisler, 1995). The circadian modulation of SWA may be a manifestation of state fragmentation, which varies as a function of circadian phase. Short episodes of sleep contain less SWA on average than long bouts, since SWA must build transiently at the start of each episode. Regardless, the independence of Process S from Process C is demonstrated by the fact that the state-dependent temporal dynamics of SWA that define process S are intact after suprachiasmatic nucleus lesions in rodents (Mistlberger et al., 1983; Tobler et al., 1983; Trachsel et al., 1992). Hence, for the purpose of addressing the quantitative relationship between sleep state and lactate concentration in the cerebral cortex, the current study assumed that process C does not regulate sleep state-dependent lactate concentration dynamics in the cerebral cortex.

Unlike SWA in the EEG, which in Process S modeling is only measured in SWS, lactate concentration can be monitored continuously throughout recording, regardless of state. The homeostatic model was thus fitted to lactate sensor data from wake, REMS and SWS; the data demonstrate asymptotic curves for the dynamics of the sensor in wake and sleep alike. These temporal dynamics of lactate concentration are such that it resembles a sleep substance. Just as SWS need (measured either by SWA amplitude in SWS or the propensity to undergo wake-to-SWS transitions) increases as a function of time spent awake or in REMS, the concentration of lactate in the brain increases as a function of time spent awake. Just as sleep need decreases as a function of time spent in SWS, the concentration of lactate in the brain declines as a function of time spent asleep. Additionally, the rate of decline of lactate concentration during SWS is a function of the depth of sleep (measured by SWA; Wisor et al., 2013). These data collectively established a relationship between SWA and sleep-dependent lactate concentration decline and led us to hypothesize (Wisor et al., 2013) that lactate concentration is a potential driver of slow wave propensity during SWS.

How might lactate directly regulate sleep-related electrophysiological events? Lactate suppresses neuronal spiking in cortical (Bozzo et al., 2013) and hippocampal (Gilbert et al., 2006) cultures and promotes network inhibition via potentiation of GABAergic signaling (Holmgren et al., 2010) in primary cortical cultures. One mechanism for the effects of lactate concentration on neuronal activity is the activation of a G-protein coupled receptor, GPR81, selectively by lactate. GPR81 activation suppresses cAMP formation and thus suppresses protein kinase A, which would be expected to reduce excitability. The suppression of spiking in cortical cultures by lactate is dependent on G protein activation and is mimicked by the GPR81 agonist 3,5-dyhydroxybenzoic acid (3,5-DHBA) (Bozzo et al., 2013). GPR81 activation provides a means whereby lactate production might dampen neuronal excitability, priming cortical networks to undergo slow waves.

Using this logic, we reasoned that lactate concentration and SWA might exhibit similar temporal dynamics when modeled homeostatically across sleep/wake cycles. But as shown here, the time constants for lactate homeostasis (τ_*i*_ and τ_*d*_) were reduced significantly compared to those of SWA. These distinct temporal dynamics render the hypothesis that SWA is a direct function of lactate concentration untenable: lactate concentration approaches asymptote values within less than an hour whether increasing or decreasing, while SWA drive continues to build over periods of hours of wakefulness. While lactate concentration *per se* might not be the biochemical determinant of sleep SWA homeostatic dynamics, it remains possible that other cellular effects of glycolysis impact SWA. The biosensors measure extracellular lactate concentration, not lactate synthesis. Extracellular lactate concentration may reach an equilibrium if the rate of clearance by transporters comes to equal that of synthesis (reviewed in Brooks, 2009). At least two other biochemical changes related to lactate synthesis but not yet subjected to homeostatic modeling are potentially relevant to sleep regulation. First, methylglyoxal is an essential byproduct of the synthesis of lactate via glycolysis. Intracerebroventricular infusion of methylglyoxal promotes sleep (Jakubcakova et al., 2013). Second, ongoing lactate synthesis builds an oxidative burden, as conversion of pyruvate to lactate (reduction) is coupled to the production of NAD+ (oxidation) (Nelson and Cox, 2008). Suppression of firing of hippocampal pyramidal cells by lactate has been attributed to changes in redox status (Gilbert et al., 2006). Enforced wakefulness is characterized by a significant oxidative burden relative to sleep (Silva et al., 2004; Cirelli, 2006; Ramanathan et al., 2010; Ramanathan and Siegel, 2011). Manipulation of oxidative stress-related pathways affects sleep wake cycles (Honda et al., 1994; Inoué et al., 1995; Kimura et al., 1998; Ikeda et al., 2005). Ongoing glycolysis, even when it does not manifest as continuing elevation of lactate concentration but rather as equilibrium lactate concentration, may thus drive sleep by these mechanisms. But this possibility highlights a limitation of the use of enzymatic lactate biosensors: it will be necessary to measure the rate of lactate synthesis, and the concentrations of byproducts such as NAD+ and methylglyoxal, rather than steady-state lactate concentration, to address the role of glycolytic byproducts in sleep regulation.

During normal waking in humans, process *S* is far from reaching the upper asymptote and wakefulness has to be continually maintained for at least 30 h for the non-linearity of *S* to take effect. Therefore, a linear, rather than exponential, model is sometimes sufficient for human data with long waking periods. To determine if a linear model would better fit the SWA data in the current study we modified the process *S* model to rise linearly rather than exponentially during wakefulness or REMS and found that the residuals were 33% higher as compared to the exponential model.

The current report applies a well-known optimization algorithm to homeostatic modeling of physiological signals related to sleep. Computational demand differed dramatically between the brute force and Nelder-Mead methods. Optimization of τ_*i*_ and τ_*d*_ for 42-h (15120-epoch) recordings of EEG required 1444 s with the brute force method and just under 3 s with non-linear Nelder Mead optimization, a 500-fold difference. For the lactate signal the brute force method required 762 s on average compared to 2 s using Nelder-Mead, a difference of about 380-fold. The low computational demand associated with Nelder-Mead facilitates the incorporation of additional tunable parameters into model optimization. For instance, previous analyses of Process S used arbitrarily-defined boundaries for SWA in the EEG, typically 1–4 Hz (Borbely, 1982; Daan et al., 1984; Franken et al., 2001). In fact, the range of EEG spectral values that exhibit homeostatic dynamics (i.e., EEG spectra at which power increases during sleep as a function of prior wake duration) is broader than this range, depending on several factors including the exact placement of electrodes and the species and genetic variant thereof being studied. One could, therefore, further optimize the fit of the model by using, as a tunable parameter, the exact frequency range considered to be SWA. For instance, one could optimize the model for SWA in ranges of 0.5–4 Hz, 0.5–5 Hz, 0.5–6 Hz, 0.5–7 Hz, 1–4 Hz, 1–5 Hz, 1–6 Hz, 1–7 Hz. If one were to use the brute force method and our computational infrastructure, these eight iterations would require 1444^*^8 = 11552 s, or 192 min of computation time for a single record. The same set of iterations could be done in far less time by framing the problem as a Nelder-Mead optimization using 4 separate parameters: the SWA range lower boundary, the SWA range upper boundary, τ_*i*_ and τ_*d*_. This approach would be similar to a previous study that calculated τ_*i*_ and τ_*d*_ based on narrower frequency bands (Vyazovskiy et al., 2007), where it was found that the time constants are slower for lower frequencies. In summary, this report demonstrates that Nelder-Mead optimization can replace brute force methods in homeostatic modeling and allow for more aggressive parameter optimization within this area of inquiry.

## Conflict of interest statement

The authors declare that the research was conducted in the absence of any commercial or financial relationships that could be construed as a potential conflict of interest.

## References

[B1] Barros F. (2013). Metabolic signaling by lactate in the brain. Trends Neurosci. 36, 396–404. 10.1016/j.tins.2013.04.002 23639382

[B2] Borbely A. A. (1982). A two process model of sleep regulation. Hum. Neurobiol. 1, 195–204. 7185792

[B3] Bozzo L. Puyal J. Chatton J.-Y. (2013). Lactate modulates the activity of primary cortical neurons through a receptor-mediated pathway. PLoS ONE 8:e71721. 10.1371/journal.pone.0071721 23951229 PMC3741165

[B4] Brooks G. A. (2009). Cell-cell and intracellular lactate shuttles. J. Physiol. 587, 5591–5600. 10.1113/jphysiol.2009.178350 19805739 PMC2805372

[B5] Buchsbaum M. S. Gillin J. Wu J. Hazlett E. Sicotte N. Dupont R. M.. (1989). Regional cerebral glucose metabolic rate in human sleep assessed by positron emission tomography. Life Sci. 45, 1349–1356. 10.1016/0024-3205(89)90021-0 2796606

[B6] Cirelli C. (2006). Cellular consequences of sleep deprivation in the brain. Sleep Med. Rev. 10, 307–321. 10.1016/j.smrv.2006.04.001 16920372

[B7] Clegern W. C. Moore M. E. Schmidt M. A. Wisor J. (2012). Simultaneous electroencephalography real-time measurement of lactate concentration and optogenetic manipulation of neuronal activity in the rodent cerebral cortex. J. Vis. Exp. [Epub ahead of print]. 10.3791/4328 23271428 PMC3576421

[B8] Daan S. Beersma D. G. Borbely A. A. (1984). Timing of human sleep: recovery process gated by a circadian pacemaker. Am. J. Physiol. 246, R161–R183. 6696142 10.1152/ajpregu.1984.246.2.R161

[B9] Dash M. B. Tononi G. Cirelli C. (2012). Extracellular levels of lactate, but not oxygen, reflect sleep homeostasis in the rat cerebral cortex. Sleep 35, 909–919. 10.5665/sleep.1950 22754037 PMC3369226

[B10] Dijk D. Czeisler C. (1995). Contribution of the circadian pacemaker and the sleep homeostat to sleep propensity, sleep structure, electroencephalographic slow waves, and sleep spindle activity in humans. J. Neurosci. 15, 3526–3538. 7751928 10.1523/JNEUROSCI.15-05-03526.1995 PMC6578184

[B11] Franken P. Chollet D. Tafti M. (2001). The homeostatic regulation of sleep need is under genetic control. J. Neurosci. 21, 2610–2621. 11306614 10.1523/JNEUROSCI.21-08-02610.2001 PMC6762509

[B12] Franken P. Tobler I. Borbly A. A. (1992). Cortical temperature and EEG slow-wave activity in the rat - analysis of vigilance state related changes. Pflugers Arch. Eur. J. Physiol. 420, 500–507. 10.1007/BF00374625 1614823

[B13] Franken P. Tobler I. Borbély A. (1991). Sleep homeostasis in the rat: simulation of the time course of EEG slow-wave activity. Neurosci. Lett. 130, 141–144. 10.1016/0304-3940(91)90382-4 1795873

[B14] Gilbert E. Tang J. Ludvig N. Bergold P. (2006). Elevated lactate suppresses neuronal firing *in vivo* and inhibits glucose metabolism in hippocampal slice cultures. Brain Res. 1117, 213–223. 10.1016/j.brainres.2006.07.107 16996036

[B15] Holmgren C. Mukhtarov M. Malkov A. Popova I. Bregestovski P. Zilberter Y. (2010). Energy substrate availability as a determinant of neuronal resting potential, GABA signaling and spontaneous network activity in the neonatal cortex *in vitro*. J. Neurochem. 112, 900–912. 10.1111/j.1471-4159.2009.06506.x 19943846

[B16] Honda K. Komoda Y. Inoué S. (1994). Oxidized glutathione regulates physiological sleep in unrestrained rats. Brain Res. 636, 253–258. 10.1016/0006-8993(94)91024-3 8012809

[B17] Hu Y. Wilson G. (1997). A temporary local energy pool coupled to neuronal activity: fluctuations of extracellular lactate levels in rat brain monitored with rapid-response enzyme-based sensor. J. Neurochem. 69, 1484–1490. 10.1046/j.1471-4159.1997.69041484.x 9326277

[B18] Ikeda M. Ikeda-Sagara M. Okada T. Clement P. Urade Y. Nagai T.. (2005). Brain oxidation is an initial process in sleep induction. Neuroscience 130, 1029–1040. 10.1016/j.neuroscience.2004.09.057 15652998

[B19] Inoué S. Honda K. Komoda Y. (1995). Sleep as neuronal detoxification and restitution. Behav. Brain Res. 69, 91–96. 10.1016/0166-4328(95)00014-K 7546322

[B20] Jakubcakova V. Curzi M. L. Flachskamm C. Hambsch B. Landgraf R. Kimura M. (2013). The glycolytic metabolite methylglyoxal induces changes in vigilance by generating low-amplitude non-REM sleep. J. Psychopharmacol. 27, 1070–1075. 10.1177/0269881113495596 23828824

[B21] Kalinchuk A. Urrila A. Alanko L. Heiskanen S. Wigren H. Suomela M.. (2003). Local energy depletion in the basal forebrain increases sleep. Eur. J. Neurosci. 17, 863–869. 10.1046/j.1460-9568.2003.02532.x 12603276

[B22] Kennedy C. Gillin J. C. Mendelson W. Suda S. Miyaoka M. Ito M.. (1982). Local cerebral glucose utilization in non-rapid eye movement sleep. Nature 297, 325–327. 10.1038/297325a0 7078644

[B23] Kimura M. Kapás L. Krueger J. M. (1998). Oxidized glutathione promotes sleep in rabbits. Brain Res. Bull. 45, 545–548. 10.1016/S0361-9230(97)00441-3 9566496

[B24] Lagarias J. C. Reeds J. A. Wright M. H. Wright P. E. (1998). Convergence properties of the nelder-mead simplex method in low dimensions. SIAM J. Optimiz 9, 112–147 10.1137/S1052623496303470

[B25] Maquet P. Dive D. Salmon E. Sadzot B. Franco G. Poirrier R.. (1990). Cerebral glucose utilization during sleep-wake cycle in man determined by positron emission tomography and [18F]2-fluoro-2-deoxy-d-glucose method. Brain Res. 513, 136–143. 10.1016/0006-8993(90)91099-3 2350676

[B26] Mistlberger R. Bergmann B. Waldenar W. Rechtschaffen A. (1983). Recovery sleep following sleep-deprivation in intact and suprachiasmatic nuclei-lesioned rats. Sleep 6, 217–233. 6622879 10.1093/sleep/6.3.217

[B27] Naylor E. Aillon D. V. Barrett B. S. Wilson G. S. Johnson D. A. Johnson D. A.. (2012). Lactate as a biomarker for sleep. Sleep 35, 1209–1222. 10.5665/sleep.2072 22942499 PMC3413798

[B28] Nelder J. A. Mead R. (1965). A simplex method for function minimization. Comput. J. 7, 308–313 10.1093/comjnl/7.4.308

[B29] Nelson D. Cox M. (2008). Lehninger Principles of Biochemistry 5th Edn., Vol. 33 (New York, NY: W. H. Freeman and Company).

[B30] Ramanathan L. Hu S. Frautschy S. A. Siegel J. M. (2010). Short-term total sleep deprivation in the rat increases antioxidant responses in multiple brain regions without impairing spontaneous alternation behavior. Behav. Brain Res. 207, 305–309. 10.1016/j.bbr.2009.10.014 19850085 PMC2815069

[B31] Ramanathan L. Siegel J. M. (2011). Sleep deprivation under sustained hypoxia protects against oxidative stress. Free Radic. Biol. Med. 51, 1842–1848. 10.1016/j.freeradbiomed.2011.08.016 21907278 PMC3197752

[B32] Reich P. Geyer S. J. Karnovsky M. L. (1972). Metabolism of brain during sleep and wakefulness. J. Neurochem. 19, 487–497. 10.1111/j.1471-4159.1972.tb01358.x 4334501

[B33] Shram N. Netchiporouk L. Cespuglio R. (2002). Lactate in the brain of the freely moving rat: voltammetric monitoring of the changes related to the sleep-wake states. Eur. J. Neurosci. 16, 461–466. 10.1046/j.1460-9568.2002.02081.x 12193189

[B34] Silva R. H. Kameda S. R. Carvalho R. C. Takatsu-Coleman A. L. Niigaki S. T. Ablio V. C.. (2004). Anxiogenic effect of sleep deprivation in the elevated plus-maze test in mice. Psychopharmacology 176, 115–122. 10.1007/s00213-004-1873-z 15160262

[B35] Tobler I. Borbely A. Groos G. (1983). The effect of sleep-deprivation on sleep in rats with suprachiasmatic lesions. Neurosci. Lett. 42, 49–54. 10.1016/0304-3940(83)90420-2 6657146

[B36] Tobler I. Franken P. Jaggi K. (1993). Vigilance states, EEG spectra, and cortical temperature in the guinea pig. Am. J. Physiol. 264, R1125–R1132. 8322965 10.1152/ajpregu.1993.264.6.R1125

[B37] Trachsel L. Edgar D. Seidel W. Heller H. Dement W. (1992). Sleep homeostasis in suprachiasmatic nuclei-lesioned rats - effects of sleep-deprivation and triazolam administration. Brain Res. 589, 253–261. 10.1016/0006-8993(92)91284-L 1393593

[B38] Trachsel L. Tobler I. Borbély A. (1988). Electroencephalogram analysis of non-rapid eye movement sleep in rats. Am. J. Physiol. 255, R27–R37. 3394843 10.1152/ajpregu.1988.255.1.R27

[B39] Van D. N. S. Brine K. (1970). Effect of sleep on brain labile phosphates and metabolic rate. Am. J. Physiol. 218, 1434–1439. 4245209 10.1152/ajplegacy.1970.218.5.1434

[B40] Vyazovskiy V. Achermann P. Tobler I. (2007). Sleep homeostasis in the rat in the light and dark period. Brain Res. Bull. 74, 37–44. 10.1016/j.brainresbull.2007.05.001 17683787

[B41] Wigren H. Rytkönen K. Porkka-Heiskanen T. (2009). Basal forebrain lactate release and promotion of cortical arousal during prolonged waking is attenuated in aging. J. Neurosci. 29, 11698–11707. 10.1523/JNEUROSCI.5773-08.2009 19759316 PMC6665766

[B42] Wisor J. P. Clegern W. C. (2011). Quantification of short-term slow wave sleep homeostasis and its disruption by minocycline in the laboratory mouse. Neurosci. Lett. 490, 165–169. 10.1016/j.neulet.2010.11.034 21111032

[B43] Wisor J. P. Rempe M. J. Schmidt M. A. Moore M. E. Clegern W. C. (2013). Sleep slow-wave activity regulates cerebral glycolytic metabolism. Cereb. Cortex 23, 1978–1987. 10.1093/cercor/bhs189 22767634 PMC3707408

[B44] Wisor J. P. Schmidt M. A. Clegern W. C. (2011). Cerebral microglia mediate sleep/wake and neuroinflammatory effects of methamphetamine. Brain Behav. Immun. 25, 767–776. 10.1016/j.bbi.2011.02.002 21333736

